# Effect of the Peptide Calcium Channel Blocker ω-hexatoxin-Hv1a on Cell Death during Ischemia/Reperfusion *in vitro*

**DOI:** 10.17691/stm2023.15.1.03

**Published:** 2023-01-28

**Authors:** E.V. Iurova, E.A. Beloborodov, Yu.V. Saenko, D.E. Sugak, A.N. Fomin, S.M. Slesarev, Ye.S. Pogodina

**Affiliations:** Junior Researcher, Laboratory of Research and Development of Peptide Drugs and Vaccines, S.P. Kapitsa Technological Research Institute; Ulyanovsk State University, 42 Leo Tolstoy St., Ulyanovsk, 432017, Russia; Researcher, Laboratory of Research and Development of Peptide Drugs and Vaccines, S.P. Kapitsa Technological Research Institute; Ulyanovsk State University, 42 Leo Tolstoy St., Ulyanovsk, 432017, Russia; Leading Researcher, Laboratory of Research and Development of Peptide Drugs and Vaccines, S.P. Kapitsa Technological Research Institute; Ulyanovsk State University, 42 Leo Tolstoy St., Ulyanovsk, 432017, Russia; Junior Researcher, Laboratory of Research and Development of Peptide Drugs and Vaccines, S.P. Kapitsa Technological Research Institute; Ulyanovsk State University, 42 Leo Tolstoy St., Ulyanovsk, 432017, Russia; Senior Researcher, Director of the S.P. Kapitsa Technological Research Institute; Ulyanovsk State University, 42 Leo Tolstoy St., Ulyanovsk, 432017, Russia; Professor, Head of the Department of Biology, Ecology and Nature Management, Institute of Medicine, Ecology and Physical Education; Ulyanovsk State University, 42 Leo Tolstoy St., Ulyanovsk, 432017, Russia; Head of the Laboratory of Research and Development of Peptide Drugs and Vaccines, S.P. Kapitsa Technological Research Institute Ulyanovsk State University, 42 Leo Tolstoy St., Ulyanovsk, 432017, Russia

**Keywords:** ischemia, reperfusion injury, peptide toxins, calcium channel blockers, apoptosis during reperfusion

## Abstract

Apoptosis and necrosis during reperfusion after ischemia are key mechanisms at the cellular level leading to damage. The development of pathological conditions is preceded by intracellular calcium ion overload both at the stage of ischemia and at the stage of reperfusion. In this regard, one of the strategies aimed at reducing damage during ischemia/reperfusion is associated with the use of calcium channel blockers.

**The aim of the study** was to study the effect of a peptide toxin, a calcium channel blocker ω-hexatoxin-Hv1a, on different types of epithelial cell death during *in vitro* reconstruction of ischemia/reperfusion conditions characteristic of organ transplantation.

**Materials and Methods:**

In this study, we used CHO-K1 epithelial cell culture. Changes in apoptosis, necrosis, cell index, and calcium ion concentration were assessed when modeling ischemia/reperfusion processes *in vitro* with the addition of a calcium channel blocker toxin. Ischemic and reperfusion injury was achieved by oxygen and nutrient deprivation followed by reperfusion in a complete nutrient medium. The measurements were performed using a multimodal plate reader-fluorimeter.

**Results:**

An increase in apoptosis, necrosis, and the concentration of calcium ions was recorded when modeling ischemia/reperfusion processes. A decrease in the level of apoptosis and necrosis, as well as the concentration of calcium ions to a physiological level or a level close to physiological, was noted when the toxin was added at a concentration of 50 nM at the reperfusion stage. The cell index showed a faster restoration in the presence of the toxin.

**Conclusion:**

The experimental data confirm the hypothesis of a beneficial effect of peptide calcium channel blockers on the state of epithelial cells during reperfusion after ischemia and can be considered for further study as a strategy for organ adaptation before reperfusion.

## Introduction

Ischemic and reperfusion injury (IRI) is an important factor leading to high morbidity and mortality in a number of conditions, which, in addition to myocardial infarction [1, 2], ischemic stroke [3, 4], and acute kidney injury [5, 6], include organ transplantation [7, 8], as well as extensive surgery. In all these cases, IRI significantly affects the clinical outcome [9]. Similar mechanisms of damage caused by IRI are observed in kidney, liver, and lung transplantation. A number of studies have shown that the epithelial and endothelial cells of transplanted organs are the first ones to be damaged [10-13].

Grafts inevitably experience ischemia from the moment they are separated from the donor blood. During transplantation, a number of sequential events are noted: after a period of surgical thermal ischemia during the removal of a donor organ, a long period of cold ischemia in a hypothermic preservative solution follows, which ends with thermal ischemia during implantation in the recipient [14]. Paradoxically, subsequent reperfusion does not restore the normal state, but enhances further the damage by activating several mechanisms, including the innate and adaptive immune response, and cell death programs [9].

At the cellular level, two phases of IRI should be distinguished: damage that occurs during ischemia and damage that occurs after reperfusion, which are characterized by cell death such as apoptosis and necrosis [15, 16]. Apoptosis has been identified as a central mechanism in many aspects of organ and tissue transplantation [17].

The first change caused by ischemia is a decrease in oxygen delivery. This causes a switch from aerobic to anaerobic metabolism. Anaerobic metabolism does not meet the needs of aerobic tissues, and, as a result, the level of intracellular adenosine triphosphate (ATP) falls rapidly, causing intracellular acidosis. All this leads to inhibition of Na/K-ATPase activity, which causes an increase in the concentration of sodium ions and water inside the cell. Along with the accumulation of Na+ ions in the cell, the level of Са^2+^ also increases since the Na/ Ca exchanger stops pumping calcium out of the cell. Since the sodium accumulated inside the cell cannot be removed by Na/K-ATPases, the Na/Ca exchanger starts to work in reverse mode [18]. Calcium overload causes the activation of calcium-dependent proteases such as calpains. They remain inactive due to the acidic medium, but can damage cells after pH normalization during reperfusion [19]. Another effect of Ca^2+^ overload is the formation of reactive oxygen species (ROS) at the mitochondrial level during ischemia [20].

Despite all of the above processes, only a small number of cells are lost during ischemia compared to the reperfusion phase. During reperfusion, an increase in the level of oxygen occurs, as well as normalization of extracellular pH. And after reperfusion, a further increase in cytoplasmic and mitochondrial calcium, which activates calpains causing disruption of the cellular structure and cell death, goes on. A return to normoxia contributes to an increased ROS production and a decrease in the level of antioxidant capacity [21, 22]. ROS, in turn, contribute to membrane and cytoskeleton damage [23]. An increase in ROS and an increased calcium content in mitochondria cause the opening of the mitochondrial pore, which leads to cell death through various mechanisms, such as apoptosis and necrosis [16, 24].

To ensure the best functioning of an organ after transplantation, two methods of organ preservation are currently used in clinical practice: static storage in a refrigerator and machine perfusion [25]. The use of several therapeutic gases, including hydrogen (H_2_), NO, hydrogen sulfide (H_2_S) and carbon monoxide [26], as well as treatment with pharmacological doses of prolyl hydroxylase inhibitors [27] and the enzyme superoxide dismutase [28], have been investigated additionally. However, since calcium overload is one of the most important pathophysiological mechanisms of IRI, the main strategy aimed to reduce damage seems to be the use of calcium channel blockers. A number of studies have found out that the use of verapamil, diltiazem, or nifedipine improves the integrity of the transplanted organ [29] and also reduces experimental ischemic and reperfusion injury of various organs [30-32] by decreasing cellular permeability for calcium.

Currently, arthropod peptide toxins belonging to the group of neurotoxins with disulfide bridges (knottins) that form an inhibitory cystine knot are considered as an alternative to chemical calcium channel blockers, the use of which has a number of significant disadvantages [33]. These peptides contain approximately 30 amino acid residues, including conserved cysteines that form intramolecular disulfide bonds [34]. Not only do most of these peptides have selectivity for a class of ion channels, they can have selectivity for a channel: from weak to exclusive [35]. In addition, the cystine knot converts toxins into hyperstable mini-proteins with great chemical, thermal, and biological stability. Knottins are generally resistant to pH extremes, organic solvents, and high temperatures. However, from a therapeutic point of view, their most important property is protease resistance [36, 37].

At present, the effect of similar toxins in the regulation of IRI has already been demonstrated. For example, one of the β-conotoxins is MVIIC, which consists of 26 amino acids and is a member of the calcium channel blocking toxin family. *In vitro* studies simulating brain and spinal cord ischemia have shown that MVIIC significantly reduces Ca^2+^ influx and attenuates glutamate release [38]. *In vitro* experiments have also shown that GVIA β-conopeptide inhibits excessive glutamate release during ischemia by blocking the N-type Ca^2+^ channel, and that this inhibition causes a significant protective effect in neurons [39].

Thus, the strategy for the use of peptide calcium blockers will be using them as a promising basis for the development of therapeutic drugs the action of which is aimed at reducing the negative consequences of reperfusion in organ transplantation.

**The aim of the study** was to study the effect of a peptide toxin, the calcium channel blocker ω-hexatoxin-Hv1a [PDB: P56207] from the spider *Hadronyche versuta*, on different types of epithelial cell death during *in vitro* reconstruction of ischemia/reperfusion conditions characteristic of organ transplantation.

## Materials and Methods

The study was carried out on the CHO-K1 culture, Chinese hamster ovary epithelial cells, since there is evidence that epithelial cells are primarily affected during ischemia/reperfusion [10-12]. No human/animal was directly involved in the sampling process during this study. Russian regulations do not require the approval of the use of cell line biomaterials for scientific research (Federal Law No.180-FZ of June 23, 2016).

The cell line was maintained in the DMEM/F12 medium (PanEco, Russia) supplemented with 10% fetal bovine serum (FBS) (Biosera, France) and gentamicin at 37°C and 5% CO_2_ in a CO_2_ incubator (Sanyo, Japan). Passages were made 24 h before the experiment in 48-well plates at a concentration of 40,000 cells per well.

The ischemia/reperfusion model was reconstructed during 3-hour cultivation of these cells in the DMEM medium (PanEco) with a reduced content of FBS (1%) and glucose (1 g/L) in 1% O_2_ and 5% CO_2_ (ischemia) in a CB-53 incubator (Binder, Germany) followed by 3-hour incubation in DMEM containing 10% FBS and 3.151 g/L glucose with 18.6% O_2_ and 5% CO_2_ (reperfusion) — control [40]. The toxin at final concentrations of 10 and 50 nM was added at the start of reperfusion. For general control, parameters were recorded under normal conditions (DMEM with 10% FBS and 3.151 g/L glucose, 18.6% O_2_, 5% CO_2_). Before the start of each experiment, the nutrient media were equilibrated under necessary conditions for 30 min.

The amino acid sequence of the ω-hexatoxin-Hv1a toxin was found using the UniProt database (P56207). The toxin was obtained on the basis of solid-phase peptide synthesis on a ResPep SL peptide synthesizer (Intavis, Germany) in accordance with a standard protocol. Peptide analysis was performed using high-performance liquid chromatography on an LC-20AD XR chromatographic system (Shimadzu, Japan) equipped with an SPD-20A spectrophotometric detector. Mass spectrometric analysis was performed on an AUTOFLEX mass spectrometer (MICROFLEX modification) (Bruker Daltonics GmbH, Germany).

During the experiment, changes in the level of apoptosis, necrosis, and calcium were recorded. Yo-Pro 1 and PI dyes (final concentration of 1 μM) [41] were used to measure the level of apoptosis and necrosis, Rhod 2 AM (1 μM) [42] was used to measure the level of calcium ions. The dyes were added 3 h after the start of reperfusion, and before it started they were used for calcium ions. After staining, all wells were washed twice with warm phosphate-buffered saline (PBS).

The measurements were performed using a CLARIOstar Plus multimodal plate reader (BMG LABTECH, Germany) in PBS in the matrix scan mode (10×10). After the experiment, the cells were washed off the plate and their concentration was calculated in a Goryaev chamber. Primary processing was performed using the MARS software (BMG LABTECH, Germany) followed by processing in Excel. All data were recalculated per 100,000 cells.

In addition, cell survival was analyzed under ischemia/reperfusion and under the action of toxins. For this, a 16-well plate system xCELLigence RTCA S16 (Agilent Technologies, USA) was used [43]. 10,000 cells were added to each well of a 16-well plate, and cell culture parameters were measured in real time. Upon reaching the exponential growth stage (after 24 h), the culture was transferred to the conditions described above.

Each experiment was performed three times in three repetitions. The results are presented as mean ± standard deviation (M±SD). The asymmetry and kurtosis criteria were used to determine the nature of the distribution. To assess the statistical significance of differences (due to a small sample size), the Mann– Whitney test was used; processing was performed in the Origin software (OriginLab, USA). Since the toxin exposure data were compared with the control conditions without a toxin, as well as with normal conditions, the Bonferroni test was used to eliminate the effect of multiple comparisons, differences between groups were considered statistically significant at p≤0.01.

## Results

**Figure 1.**
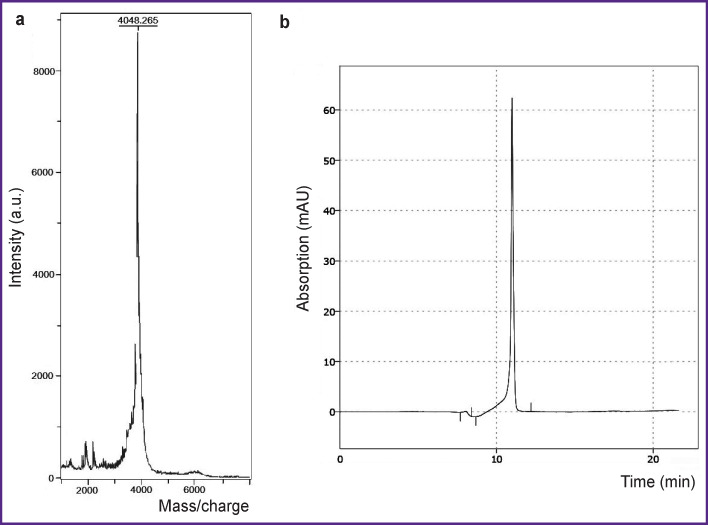
Mass spectrogram (a) and chromatogram (b) of the ω-hexatoxin-Hv1a toxin a.u. — arbitrary unit; mAU — milli absorbance unit

As a result of peptide synthesis, a toxin with a purity of >95% was obtained (Figure 1).

**Figure 2.**
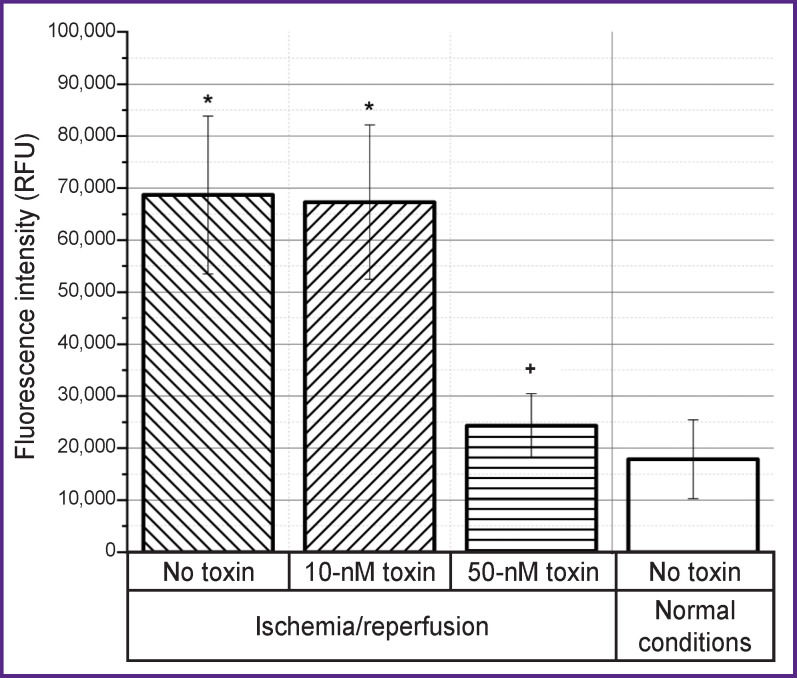
Effect of the ω-hexatoxin-Hv1a toxin at concentrations of 10 and 50 nM on the level of apoptosis after 3 h of reperfusion after ischemia ^+^ differences are statistically significant compared to the group without the toxin, p<0.01; * compared to normal conditions, p<0.01; RFU — relative fluorescent unit

A change in the level of apoptosis under the action of the toxin at concentrations of 10 and 50 nM during modeling of ischemia/reperfusion conditions showed that after 3-hour reperfusion in the CHO-K1 culture (control), an increase in the level of apoptosis occurs, however, the addition of the toxin at a concentration of 50 nM maintains the state of the culture at the level of normal conditions (Figure 2). At the same time, the toxin at a concentration of 10 nM does not affect the level of apoptosis.

**Figure 3.**
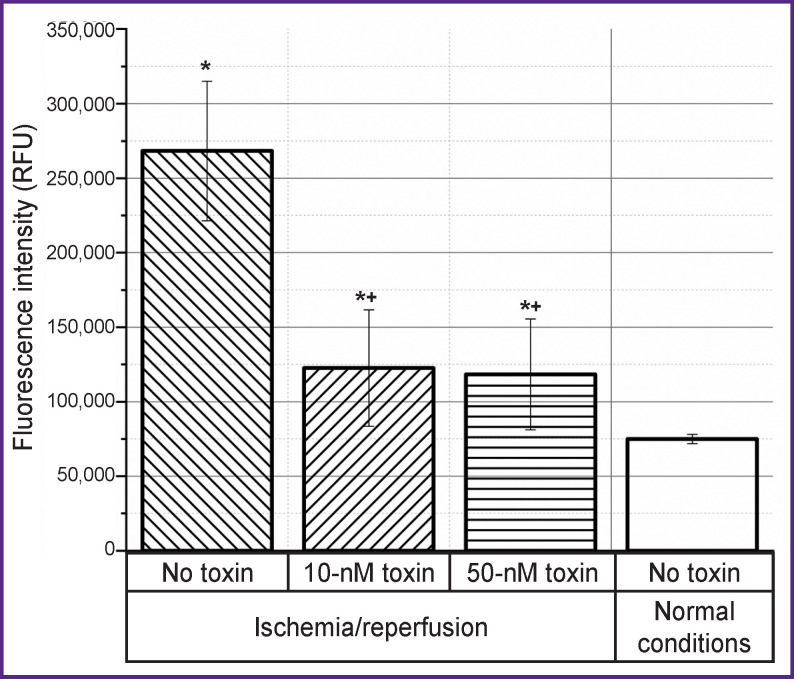
Effect of the ω-hexatoxin-Hv1a toxin at concentrations of 10 and 50 nM on the level of necrosis after 3 h of reperfusion after ischemia ^+^ differences are statistically significant compared to the group without the toxin, p<0.01; * compared to normal conditions, p<0.01; RFU — relative fluorescent unit

A similar picture is observed when studying the effect of the toxin on the level of necrosis when modeling ischemia/reperfusion conditions (Figure 3). After reperfusion, the level of necrosis also increases, but the toxin already at a concentration of 10 nM contributes to maintaining this level, which corresponds to the level of necrosis under normal conditions.

**Figure 4.**
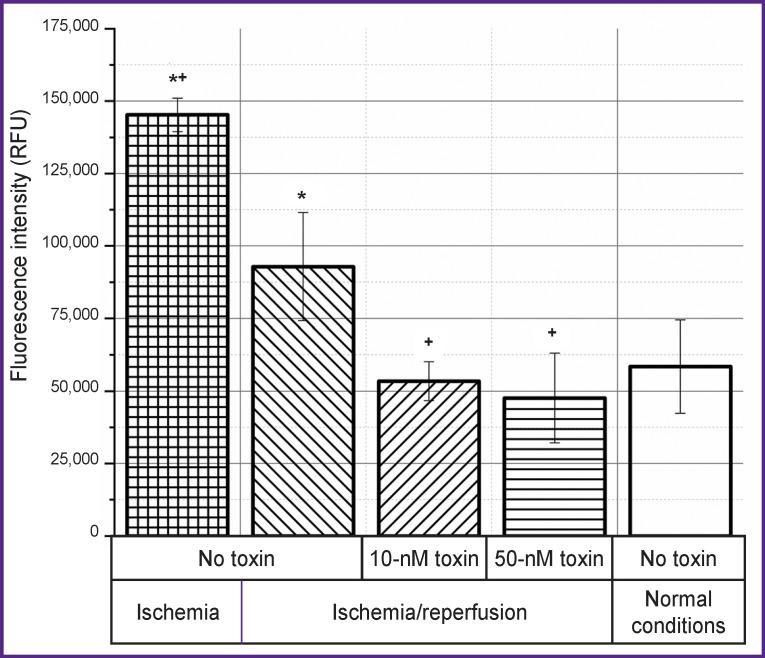
Effect of the ω-hexatoxin-Hv1a toxin at concentrations of 10 and 50 nM on the concentration of calcium ions after 3 h of reperfusion after ischemia ^+^ differences are statistically significant compared to the group without the toxin, p<0.01; * compared to normal conditions, p<0.01; RFU — relative fluorescent unit

In our experiments, an increase in the level of apoptosis and necrosis is accompanied by an increase in the concentration of calcium ions (Figure 4). At the same time, the calcium concentration increases significantly immediately after ischemia, and after 3 h of reperfusion it decreases, but remains at an above-normal conditions level. The addition of the toxin in this case leads to a decrease in the concentration of calcium ions during reperfusion, which correlates with the data on a decrease in the level of apoptosis and necrosis with the addition of the toxin. The effect is observed both at a toxin concentration of 10 and 50 nM.

**Figure 5.**
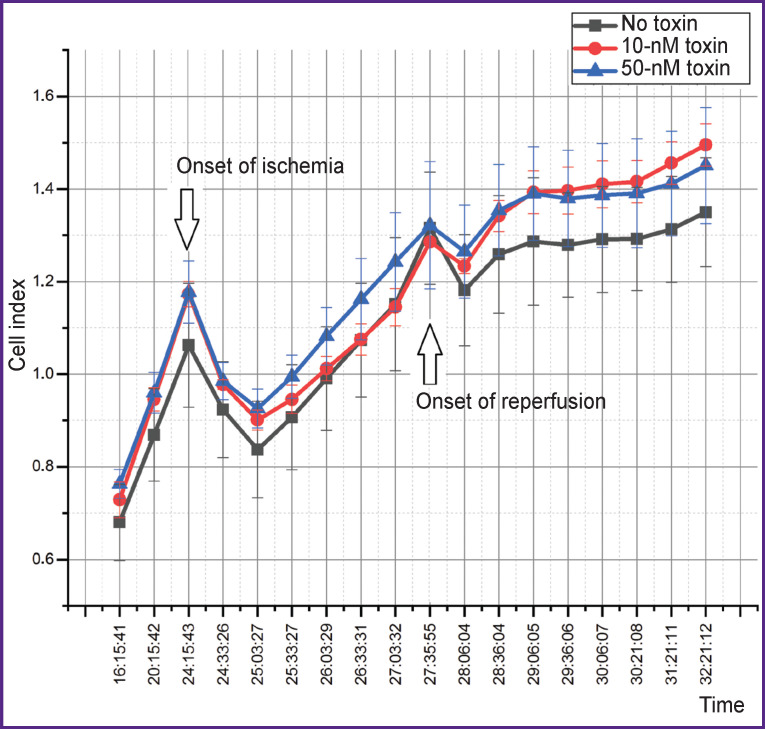
Change in the cell index under ischemia/reperfusion with and without the addition of the toxin

The dynamics of the cell index (Figure 5), which indicates a change in cell adhesion under various forms of exposure, should be considered separately. At the beginning of ischemia, the index has been found to fall within the first 30 min (the stage of adaptation to a change in the medium), then, after the normalization of conditions, the index begins to rise and continues to rise during the entire time of incubation under hypoxic conditions with serum and glucose deprivation. At the beginning of reperfusion, the index falls again due to a change in the medium, and then also begins to rise. However, in the presence of the toxin at both concentrations, the index returns to the values before the start of reperfusion already after 1 h. Under the condition when the toxin is not added to the medium, the index remains below the initial value throughout further incubation and reaches the normal level only 5 h after reperfusion starts.

## Discussion

Ischemic and reperfusion injury is a pathological condition characterized by an initial decrease in the blood supply to an organ, followed by reperfusion and reoxygenation [9]. Ischemia-based IRI followed by reperfusion occurs in all transplanted organs in a more or less severe form [44]. A significant part of damage to the ischemic organ develops not during ischemia, but during and after reperfusion and leads primarily to the dysfunction of the epithelial cells of the renal tubules [10, 17], as well as the epithelium of the bile ducts [45], contributing to the development of acute injury, delayed graft functioning, as well as acute and chronic organ rejection [13].

In our experiments, the induction of cell death was studied by modeling the processes of hypoxia–deprivation/reoxygenation–reperfusion, which characterize many pathological conditions associated with ischemic and reperfusion injury, including during transplantation. We used a model based not only on hypoxia, but also on the restriction of the nutrient access to cells. The ω-hexatoxin-Hv1a toxin was used as a calcium channel blocker; its effectiveness in reducing apoptosis induced by the proapoptotic peptide at concentrations of 10 and 50 nM had already been shown in our previous work [46].

Reperfusion injury as an effector phase of ischemia develops within hours or days after ischemia. The processes of repair and regeneration of the organ proceed along with cell apoptosis, autophagy, and necrosis; organ survival depends on whether cell death or regeneration predominates. Since apoptosis requires energy and protein synthesis, it occurs mainly during reperfusion. When cytosolic calcium, which increases during ischemia, returns to normal, cells can recover from injury. However, a progressive increase in the calcium content in the cytosol indicates an irreversible phase of damage [19]. Similar events could be seen in our experiments. At the reperfusion stage after ischemia, both apoptosis and necrosis increase (see Figures 2 and 3). At the same time, reperfusion is accompanied by an increase in the concentration of calcium ions, which begins to increase already at the stage of ischemia (see Figure 4). Mitochondrial dysfunction is a critical event during ischemia because it initiates both necrosis and apoptosis cascades during reperfusion. The organelle is both a site for the formation of harmful particles and a target for injury. During ischemia, the Na/Ca antiporter stops pumping calcium out of the cell, since the sodium accumulating inside the cell cannot be removed by Na/ K-ATPases, which leads to the Na/Ca exchanger starting to work in reverse mode [18]. Calcium overload in mitochondria causes early inhibition of NADH-coenzyme Q-oxidoreductase (complex I) with subsequent oxidation of cytochrome A3. All this leads to cytochrome C losing its binding to the inner mitochondrial membrane and penetrating into the cytosol, activating caspase 3 [47].

The addition of a calcium channel blocker toxin leads to a decrease in the calcium concentration, which contributes to the inhibition of the processes of apoptosis and necrosis during reperfusion. Similarly, this result is confirmed by the data on changes in the cell index, which shows a degree of cell spreading and their response to changing conditions (see Figure 5). In the group where the toxin was not used, the return to the normal state of the cell culture occurs after several hours, while when the toxin is added, this process takes 1 h.

## Conclusion

The experimental data confirm the hypothesis of a beneficial effect of calcium channel blockers on the state of epithelial cells during reperfusion after ischemia and can be considered for further study as a strategy for using them for organ adaptation before reperfusion. A potential way to apply a similar strategy may be associated with the preliminary storage of the transplanted organ in a solution containing nanomolar concentrations of the toxin.
